# Distribution of testate amoebae in bryophyte communities in São Miguel Island (Azores Archipelago)

**DOI:** 10.3897/BDJ.9.e63290

**Published:** 2021-03-17

**Authors:** Martin Souto Souto, Vítor Gonçalves, Xabier Pontevedra-Pombal, Pedro M. Raposeiro

**Affiliations:** 1 CIBIO, Centro de Investigação em Biodiversidade e Recurso Genéticos – Polo dos Açores, InBio, Laboratório Associado / Universidade dos Açores, Ponta Delgada, Portugal CIBIO, Centro de Investigação em Biodiversidade e Recurso Genéticos – Polo dos Açores, InBio, Laboratório Associado / Universidade dos Açores Ponta Delgada Portugal; 2 Faculty of Sciences and Technology, University of the Azores, Ponta Delgada, Portugal Faculty of Sciences and Technology, University of the Azores Ponta Delgada Portugal; 3 Dpto. Edafoloxía e Química Agrícola, Fac. Bioloxía, Universidade de Santiago de Compostela, Santiago de Compostela, Spain Dpto. Edafoloxía e Química Agrícola, Fac. Bioloxía, Universidade de Santiago de Compostela Santiago de Compostela Spain

**Keywords:** biodiversity, community ecology, island, moss, Protozoa, substratum specificity

## Abstract

**Background:**

Testate amoebae are a polyphyletic group of protists living preferentially in soils, freshwaters and wetlands. These Protozoa have a worldwide distribution, but their presence and diversity in the Azores (a remote oceanic archipelago) is poorly known, with only twelve taxa recorded so far. The published information reflects occasional collections from sporadic field visits from naturalists to São Miguel Island, mainly in the nineteenth century. To overcome this limitation, a standardised survey was carried out on the Island, sampling different types of habitats from several localities to provide the distribution and information on species ecology of testate amoebae.

**New information:**

In this study, 43 species of testate amoebae were recorded (within a total of 499 occurrences), belonging to two orders of Protista (26 Arcellinida and 17 Euglyphida). The most frequently occurring testate amoebae were *Euglypha strigosa*, *Trinema lineare*, *Euglypha rotunda*, *Assulina muscorum* and *Cyclopyxis eurystoma*. The most diverse genus was *Euglypha* (six species). A total of 38 species are new records for the Azores Archipelago. These data help to improve knowledge of the geographical distribution of testate amoebae in the northern hemisphere and their diversity in the Azores Archipelago.

## Introduction

Testate amoebae are a polyphyletic group of small size [ranging from 7 to 500 µm long (Clarke 2003)] protists, enclosed within a xenosomic or idiosomic shell or test, made from proteinaceous, calcareous and/or siliceous material (Mitchell et al. 2008), with one or two oral apertures. They have a worldwide distribution, occurring in aquatic and terrestrial systems (Smith et al. 2007). In aquatic environments, they play an important role, especially in material cycling and energy flow (Glime 2017), while in terrestrial habitats, they play a crucial role in carbon and nitrogen cycling (Puppe et al. 2015). Due to their importance and sensitivity to environmental changes in both systems, they have been frequently used as bioindicators of environmental quality or stress or ecosystem resilience (Mitchell et al. 2003, Nguyen-Viet et al. 2007, Zapata et al. 2008). In addition, their shells are usually well preserved in sedimentary records and remain nearly unchanged over time. As the species composition of these protists depends on environmental conditions, they are frequently used to reconstruct the past climate and environment (Ellison 1995, Mitchell et al. 2001, Mitchell et al. 2008). The increasing use of testate amoebae in palaeoecological studies in the last decades demands the knowledge of modern assemblages for comparative analysis (Mitchell et al. 2001, Charman 2001, Booth 2002, Swindles et al. 2015) and for the establishment of their functional traits (Mitchell et al. 2014, Amesbury et al. 2016, Marcisz et al. 2020).

Despite their great importance, current knowledge of testate amoebae in the Azores Archipelago is limited when compared to other groups (e.g. Borges et al. 2010, Raposeiro et al. 2012, Pereira et al. 2014) and previous studies are fragmented and unsystematic. Interest in Azorean testate amoebae started almost two centuries ago with the work of Ehrenberg (1854), which reported three protist species *Difflugia azorica*, *Difflugia oligodon* and *Trinema enchelys*, found in soil collected on São Miguel Island. *Difflugia azorica* was described by Ehrenberg (1871) as an endemic species, although the diagnosis can be applied to many species of the genus and may correspond to a variety of *Difflugia pyriformis*. Leidy (1879), in his work “Fresh-water rhizopods of North America”, quotes the species again, without adding any comment and *Difflugia oligodon* was described by Ehrenberg (1844) on the basis of samples from Kurdistan and a 10-word diagnosis.

Later, with the Challenger expedition that took place from 1872 to 1876 and which had a brief passage to São Miguel Island, the Irish naturalist Archer (Archer 1874) studied samples from Lake Furnas and reported six new taxa. By the end of the nineteenth century, Gerne (1888) and Barrois (1896) published several papers on freshwater biota of the Azores including some additions to testate amoebae fauna (Table 1).

Almost 100 years later, Gadea (1979), in his work on nematodes living in mosses, recorded three genera (*Centropyxis*, *Euglypha* and *Plagiostoma*). Of the three genera reported, *Plagiostoma* seems to be a misprint and refers to *Centropyxis plagiostoma* or genus *Plagiopyxis*. Since that time, no studies have been carried out on testate amoebae in the Archipelago.

The main objective of this data paper is to provide a record of the diversity and detailed distribution of testate amoebae in São Miguel Island (Azores Archipelago, Portugal). Additional information on species ecology is also discussed. Our purpose is to release this valuable dataset since no similar datasets have been previously published for Azores and it constitutes a relevant tool for comparison for ecologists studying, for example, biogeographic patterns or climate change and as modern analogues for environmental reconstructions on oceanic islands in paleoecological studies.

## Project description

### Title

Records of testate amoebae in São Miguel Island (Azores Archipelago)

### Personnel

Collections were undertaken and occurrence data recorded during 2020 in São Miguel Island. The collectors were Martin Souto, Vitor Gonçalves and Pedro Miguel Raposeiro. Identification was done by Martin Souto and Xabier Pontevedra-Pombal. Production and analysis of scanning electron microscopy images was done by Xabier Pontevedra-Pombal.

### Study area description

**Figure 1.**
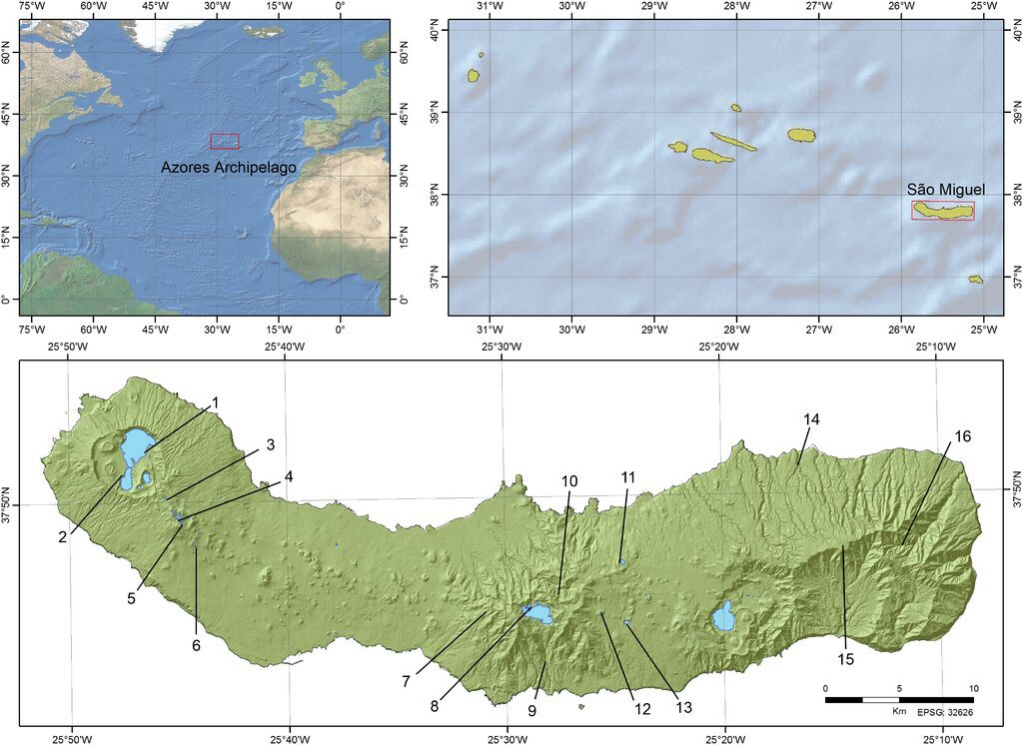
Geographical location of the study localities. At top left - The Azores Archipelago in the Atlantic Ocean highlighted by a square; at top right, São Miguel Island in the Azores Archipelago highlighted by a square; and, at the bottom, the 16 study localities.

The Azores is an oceanic archipelago located in the middle of the North Atlantic, about 1500 and 2100 km off the coast of Portugal (Europe) and North America, respectively (Fig. 1).

Native forests cover less than 10% of the total area, mostly at elevations > 800 m a.s.l. (Borges et al. 2010, DDRF 2014), being a priority habitat in the Natura 2000 network (Guimarães and Olmeda 2008). Dominant tree species of this endemic forest are *Juniperus brevifolia* (Seub.) Antoine, *Laurus azorica* (Seub.) Franco and *Ilex azorica* Loes. with a close canopy in which a great diversity of ferns and mosses is found (Elias et al. 2016). *Cryptomeria japonica* (Thunb. ex L.f.) D. Don forest occupies about 22% of the land area in the Azores (representing 60% of forest plantation area (Cruz et al. 2007), located especially at elevations > 400 m a.s.l. (DDRF 2014). These Japanese cedar forests are very dense, limiting the development of ferns and some mosses. The bryophyte communities present under the canopy of this forest is dominated by *Leucobryum juniperoideum* (Brid.) Müll. Hal., *Marchantia paleacea* Bertol., *Trichocolea tomentella* (Ehrh.) Dumort, *Thuidium tamariscinum* (Hedw.) Schimp. *and Hypnum cupressiforme* Hedw. Peatlands, mainly located in depressions in high elevation areas and cover an area of 3000 ha (Mendes and Dias 2017), are characterised by the strong development of different species of *Sphagnum* and other bryophytes (Dias and Mendes 2007). Apart from their ecological importance, peatlands are, together with lakes (e.g. Hernández et al. 2017, Raposeiro et al. 2017, Vázquez-Loureiro et al. 2019), the best paleoecological archives available in the Azores. Due to the existence of active volcanoes, São Miguel Island is particularly rich in hydrothermal vent fields (Gaspar et al. 2015). Biological communities of wetlands located close to these hydrothermal sites are influenced by higher temperatures and CO_2_-rich mineral waters. In this specific habitat, plant communities consist mainly of vascular plants, such as *Juncus effusus* L. and *Equisetum telmateia* Ehrh. Hydrothermal carbonisation of different wetland biomass wastes allows for the development of a rich community of bryophytes characterised by species that tolerate extreme conditions (Elmarsdóttir et al. 2015), such as lawn communities of *Sphagnum* spp, *Calliergon* sp. and *Polytrichum* sp. According to (Porteiro 2000), São Miguel Island has 33 lakes, located at a range of between 260 m (Azul and Verde) and 830 m in altitude (Éguas Norte). In general, the dominant vegetation of lake shores are *J. effusus*, *Osmunda regalis* L. *Agrostis* sp., surrounded by hygrophyte shrub communities of *Calluna vulgaris*. The most abundant bryophytes are *Rhytidiadelphus squarrosus* (Hedw.) Warnst, *H. cupressiforme* and several *Sphagnum* species. The presence of dense carpets of *Fissidens* sp. or *Campylopus* sp. is more common in open areas.

### Funding

This work was funded by FCT– Foundation for Science and Technology, the European Union, QREN, FEDER, COMPETE programmes (PMR - DL57/2016/ICETA/EEC2018/25; MSS - ICETA/EEC2018/25; DiscoverAzores project - PTDC/CTA-AMB/28511/2017; CIBIO/InBIO - UID/BIA/50027/2013 and POCI-01-0145-FEDER-006821) and Consolidation and Structuring project 2018 GRC-ED431C 2018/32 of the government of the Xunta Galicia.

## Sampling methods

### Study extent

This study covers 16 sampling locations on São Miguel Island (Fig. 1, Table 2), encompassing several habitat types (native forest, *Cryptomeria* forest, lake shore, peatland and hydrothermal vents). Within these habitats, different species of bryophytes were subsampled in triplicate, from a total of 46 moss subsampling locations, comprising a total of 138 samples.

### Sampling description

**Figure 2.**
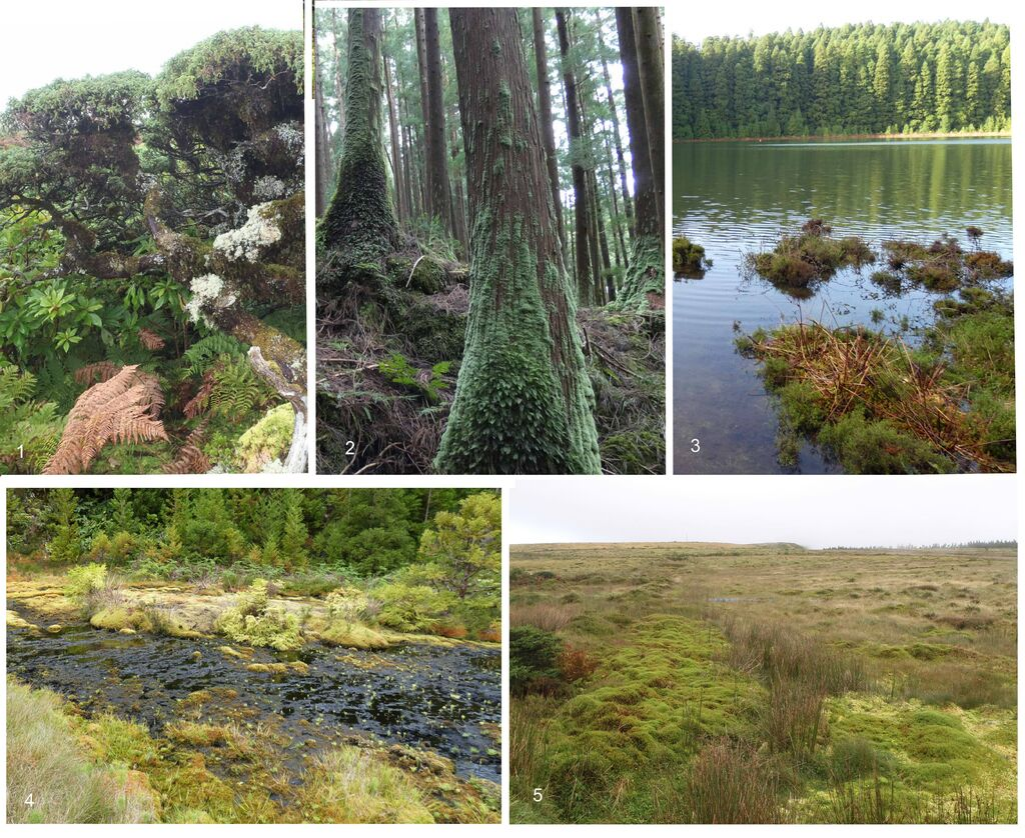
Different habitats sampled in this study: 1) native forest, 2) *Cryptomeria* forest, 3) lakeshore, 4) hydrothermal vent and 5) peatlands.

Testate amoebae collections were taken at the beginning of the growing season, between spring (February - March) and summer 2020 (May - June). In each location, several types of vegetation were chosen for their homogeneity and abundance (e.g. forest, lowland shrub, shrubland, wetland, peatland, riparian communities (Fig. 2)) and within these communities, the most abundant bryophytes were sampled. In each subsampling site, three homogeneous subplots (10 × 10 cm) were chosen, defining a total 138 sampling points (Table 2).

### Step description

Testate amoebae were sorted by fragmenting, washing and stirring of 10 × 10 cm of a wet mass of moss material into 1 litre of distilled water and then sieved through a 300 μm mesh size to remove large moss particles. The samples were concentrated by sedimentation and stored in vials with 50% alcohol at 4°C. A small aliquot of each sample was stored as a reference collection.

A drop of each sample (three subplots) was mounted on a semi-permanent slide and all testate amoebae were identified at 200x and 400x magnification using a compound microscope Leica DM2500. All measurements were made on photomicrographs (Leica DFC495 camera) of at least than 10 specimens, using image analysis software (Leica Application Suite version 3.8.0).

In order to obtain Scanning Electronic Microscopy (SEM) images, an aliquot was dried on aluminium supports with a carbon film. They were metallised with Iridium (40 nm) in a BioRad Microscience ion plating system and examined in a Zeiss Ultra Plus field emission microscope, at 5 kV high electric voltage.

The identification of testate amoebae was based on Ogden and Hedley 1980, Todorov and Bankov 2019. The classification at higher ranks follows Adl et al. (2019). Identification of the bryophyte species follow Smith and Smith (2004). Accepted names and authorities for vascular plants and bryophytes were checked in http://www.theplantlist.org (June 2020). Comparison of species richness (S) amongst different habitats was tested using one-way analysis of variance (ANOVA). Tukey’s honest significant difference (HSD) test was used as the multiple comparison post-hoc test when significant differences were identified in the ANOVA.

## Geographic coverage

### Description

The Azores is an oceanic archipelago located in the middle of the North Atlantic, about 1500 and 2100 km off the coast of Portugal (Europe) and North America, respectively (Fig. 1). The Archipelago is comprised of nine volcanic islands that are divided into three groups: the western group (Corvo and Flores Islands), the central group (Faial, Pico, Graciosa, São Jorge and Terceira Islands) and the eastern group (São Miguel and Santa Maria Islands). São Miguel is the largest Island, with an area of 746 km² and approximately 45% of the Island is between 300-800 m a.s.l., with a maximum elevation of 1103 m. The climate in the Azores is temperate oceanic, with regular and abundant rainfall, with high levels of relative humidity (up to 95% in high elevation native forests), ensuring moderate thermal variations throughout the year (Brito de Azevedo et al. 1999). Mean annual temperatures range between 14 and 18ºC, while the mean annual precipitation is between 740 and 2400 mm (Marques et al. 2008, Hernández et al. 2016).

### Coordinates

37.704 and 37.917 Latitude; -25.857 and -25.125 Longitude.

## Taxonomic coverage

### Description

Testate amoebae found on São Miguel Island

### Taxa included

**Table:** 

Rank	Scientific Name	
order	Arcellinida	
family	Arcellidae	
species	*Arcella arenaria* Greeff, 1866	
species	*Arcella catinus* Penard, 1890	
family	Netzeliidae	
species	*Cyclopyxis eurystoma* Deflandre, 1929	
species	*Cyclopyxis kahli* Deflandre, 1929	
infraorder	*Incertae sedis* Sphaerothecina	
species	*Trigonopyxis arcula* (Leidy, 1879) Penard, 1912	
family	Difflugiidae	
species	*Difflugia bacillifera* Penard, 1890	
species	*Difflugia glans* Penard, 1902	
species	*Difflugia elegans* Penard, 1890	
family	Centropyxidae	
species	*Centropyxis aerophila* Deflandre, 1929	
species	*Centropyxis constricta* (Ehrenberg, 1841) Penard, 1890	
species	*Centropyxis discoides* (Penard, 1890) Deflandre, 1929	
species	*Centropyxis elongata* (Penard, 1890) Tomas, 1959	
family	Hyalospheniidae	
species	*Alabasta militaris* (Penard, 1890) Duckert, Blandenier, Kosakyan & Singer, 2018	
species	*Nebela collaris* (Ehrenberg, 1848) Leidy, 1879	
species	*Padaungiella lageniformis* (Penard, 1890) Lara & Todorov, 2012	
species	*Padaungiella tubulata* (Brown, 1911) Lara & Todorov, 2012	
species	*Planocarina carinata* (Archer, 1867) Kosakyan et al., 2016	
species	*Quadrulella symmetrica* (Wallich, 1863) Kosakyan et al., 2016	
family	Heleoperidae	
species	*Heleopera rosea* Penard, 1890	
species	*Heleopera sphagni* Leidy, 1874	
family	Microchlamyidae	
species	*Pyxidicula cymbalum* Penard, 1902	
family	Phryganellidae	
species	*Phryganella acropodia* (Hertwig & Lesser, 1874) Hopkinson, 1909	
family	Cryptodifflugiidae	
species	*Cryptodifflugia oviformis* Penard, 1890	
order	*Incertae sedis* Arcellinida	
species	*Argynnia caudata* (Leidy, 1879)	
species	*Argynnia dentistoma* (Penard, 1890)	
species	*Physochila griseola* Penard, 1911	
order	Euglyphida	
family	Euglyphidae	
species	*Euglypha acanthophora* (Ehrenberg, 1841) Perty,1849	
species	*Euglypha cristata* Leidy, 1874	
species	*Euglypha filifera* Penard, 1890	
species	*Euglypha laevis* (Ehrenberg, 1845) Perty, 1849	
species	*Euglypha rotunda* Wailes, 1911	
species	*Euglypha strigosa* (Ehrenberg, 1871) Leidy, 1879	
species	*Tracheleuglypha dentata* (Vejdovsky, 1882) Deflandre, 1928	
family	Assulinidae	
species	*Assulina muscorum* Greeff, 1888	
family	Cyphoderiidae	
species	*Cyphoderia ampulla* (Ehrenberg, 1840) Leidy, 1879	
family	Sphenoderiidae	
species	*Sphenoderia fissirostris* Penard, 1890	
species	*Trachelocorythion pulchellum* (Penard, 1890) Bonnet, 1979	
family	Trinematidae	
species	*Corythion constricta* (Certes, 1889) Jung, 1942	
species	*Corythion dubium* Taranek, 1881	
species	*Playfairina valkanovi* Golemansky, 1966	
species	*Trinema complanatum* Penard, 1890	
species	*Trinema enchelys* (Ehrenberg, 1838) Leidy, 1879	
species	*Trinema lineare* Penard, 1890	

## Traits coverage

### Data coverage of traits

PLEASE FILL IN TRAIT INFORMATION HERE

## Temporal coverage

### Notes

04-02-2020 through to 22-06-2020

## Usage licence

### Usage licence

Open Data Commons Attribution License

### IP rights notes

This work is licensed under a Creative Commons Attribution (CC-BY) 4.0 Licence.

## Data resources

### Data package title

Checklist of testate amoebae in São Miguel Island (Azores Archipelago)

### Resource link

http://ipt.gbif.pt/ipt/resource?r=tecamebas

### Alternative identifiers

https://www.gbif.org/dataset/13c79ceb-0ceb-424f-b000-7991d7c49834

### Number of data sets

1

### Data set 1.

#### Data set name

Checklist of testate amoebae in São Miguel Island (Azores Archipelago)

#### Data format

Darwin Core Archive

#### Number of columns

32

#### Description

This paper presents data distribution of testate amoebae in São Miguel Island (Azores Archipelago) collected during 2020. The dataset has been published as a Darwin Core Archive (DwC-A), which is a standardised format for sharing biodiversity data as a set of one or more data tables (Souto et al. 2021). The core data table contains 16 events (eventID), 499 occurrences (occurrenceID) with 43 taxa (taxonID). The number of records in the data table is illustrated in the IPT link. This IPT archives the data and thus serves as the data repository. The data and resource metadata are available for downloading in the downloads section.

**Data set 1.** 

Column label	Column description
id	Identifier of the record, coded as a global unique identifier
locality	Name of the locality where the event occurred
continent	Continent of the sampling site
country	Country of the sampling site
island	Island from the Island Group of the sampling site
islandGroup	Island group of the sampling site
eventID	Identifier of the event, unique for the dataset
occurrenceID	Identifier of the occurrence, coded as a global unique identifier
type	The nature of the resource
Habitat	Habitat sampled
basisOfRecord	The specific nature of the data record
samplingProtocol	Sampling protocol
recordedBy	Person who collected the specimens
identifiedBy	Person who identified the specimens
eventDate	Time interval when the event occurred
taxonID	The identifier for the set of taxon information (data associated with the Taxon class). Specific identifier to the dataset
scientificName	The name with authorship applied on the first identification of the specimen
Kingdom	Kingdom name
Phylum	Phylum name
Class	Class name
Order	Order name
Family	Family name
Genus	Genus name
specificEpithet	The name of the first or species epithet of the scientificName
scientificNameAuthorship	The specimen accepted name, with authorship
taxonRank	The taxonomic rank of the most specific name in the scientificName
minimumElevationInMetres	Elevation in metres
decimalLatitude	The geographic latitude of the sampling site
decimalLongitude	The geographic longitude of the sampling site
coordinateUncertaintylnMetres	The indicator for the accuracy of the coordinate location in metres, described as the radius of a circle around the stated point location.
geodeticDatum	The spatial reference system upon which the geographic coordinates are based
countryCode	Code of the country where the event occurred

## Additional information

### Analysis

This study presents 499 testate amoebae (Protista) occurrences in 46 sampled sites (16 localities) in São Miguel Island, belonging to 43 species from 25 genera, 14 families and two orders. The order Euplyphida, represented by five families, accounted for 58.5% of the total occurrences and the order Arcellinida 41.5% of the total occurrences.

**Figure 3.**
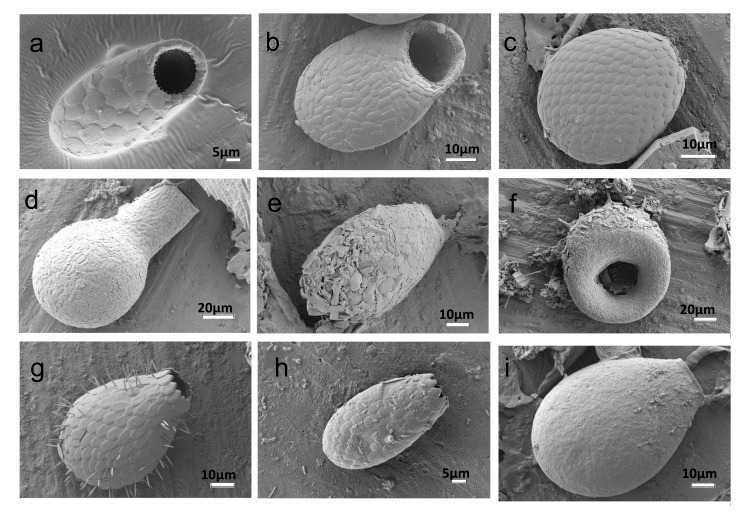
SEM images of most frequent testate amoebae found in São Miguel: a) *Trinema lineare*, b) *Corythion constricta*, c) *Assulina muscorum*, d) *Padaungiella lageniformis*, e) *Heleopera sphagni*, f) *Cyclopyxis eurystoma*, g) *Euglypha laevis*, h), *Euglypha strigosa* and i) *Nebela collaris*

**Figure 4.**
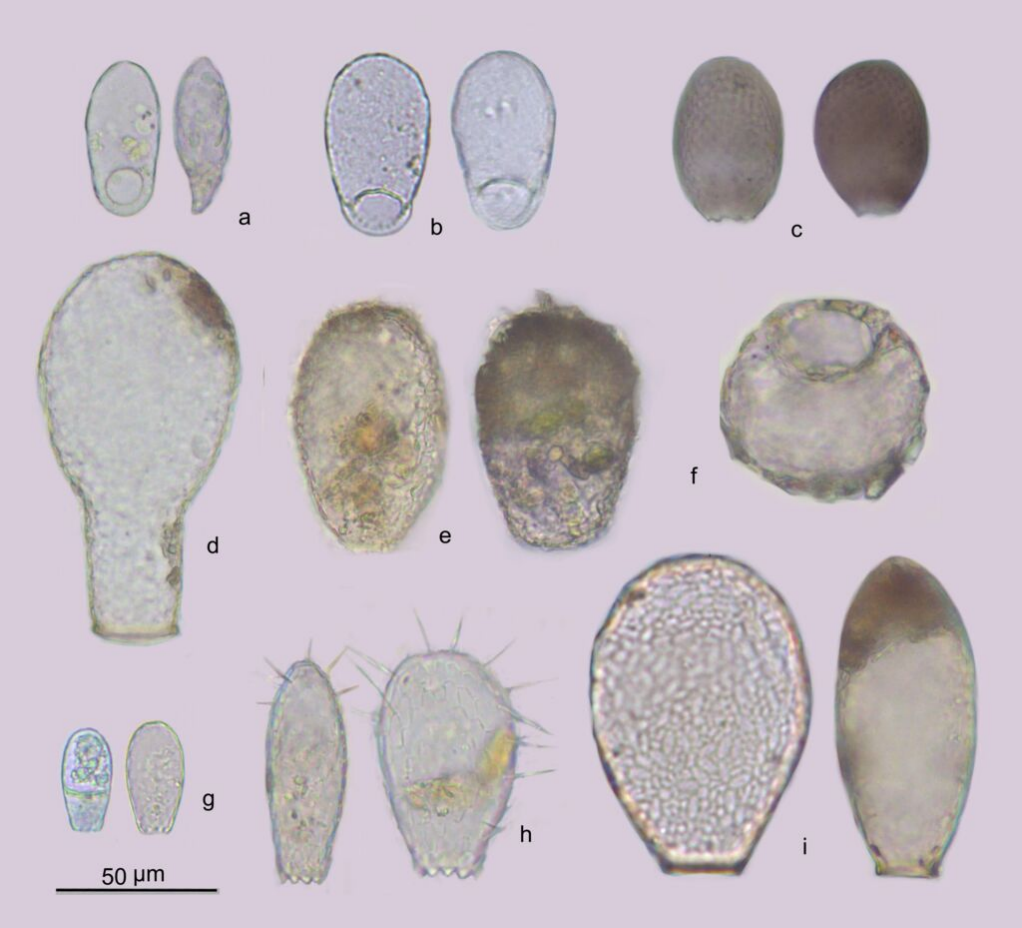
Most frequent testate amoebae found in São Miguel: a) *Trinema lineare*, b) *Corythion constricta*, c) *Assulina muscorum*, d) *Padaungiella lageniformis*, e) *Heleopera sphagni*, f) *Cyclopyxis eurystoma*, g) *Euglypha laevis*, h), *Euglypha strigosa* and i) *Nebela collaris*.

The families with the highest number of occurrences were Euglyphidae and Trinematidae (121), Hyalospheniidae (54), Heleoperidae (38), Centropyxidae (35) and Assulinidae (32). Additionally, the families with the highest number of taxa were Euglyphidae and Trinematidae (7), followed by Hyalospheniidae (6) and Centropyxidae (5). The families with lower occurrences (< 5) were Cyphoderiidae (4) and Microchlamyidae (1). The genera with the highest number of occurrences were *Euglypha* (106), *Trinema* (82), *Heleopera* (38) and *Centropyxis* (35). The other 21 genera had less than 35 occurrences. The genera with the highest number of taxa were *Euglypha* (6) and *Centropyxis* (4). *Euglypha strigosa, Trinema lineare* and *Euglypha rotunda* were the most frequent species occurring in 39, 37 and 34 sites, respectively. *Assulina muscorum* (32 sites), *Cyclopyxis eurystoma* (27 sites), *Corythion dubium* (26 sites), *Trinema complanatum* (25 sites) and *Euglypha laevis* (24 sites) were amongst the most ubiquitous testate amoebae (Figs 3, 4).

**Figure 5.**
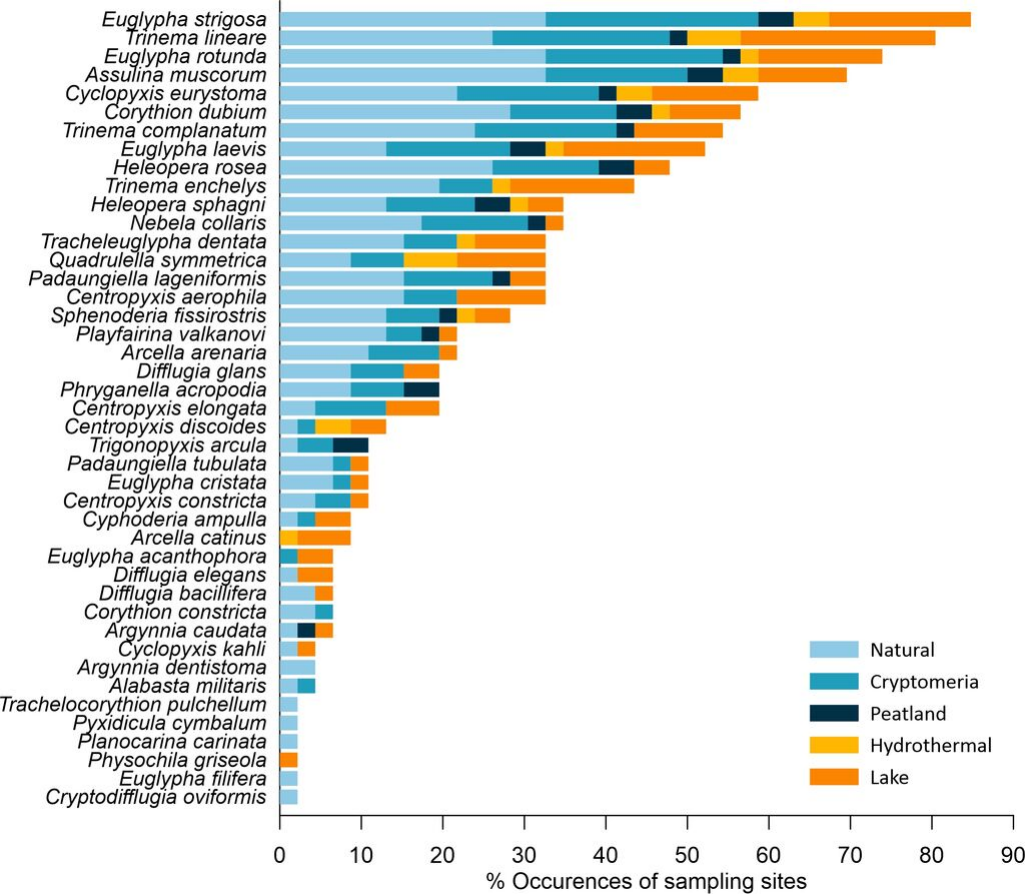
Percentage of occurrence of each species for the 46 samples and their distribution amongst habitats (proportion of occurrences is represented in colour bars)

**Figure 6.**
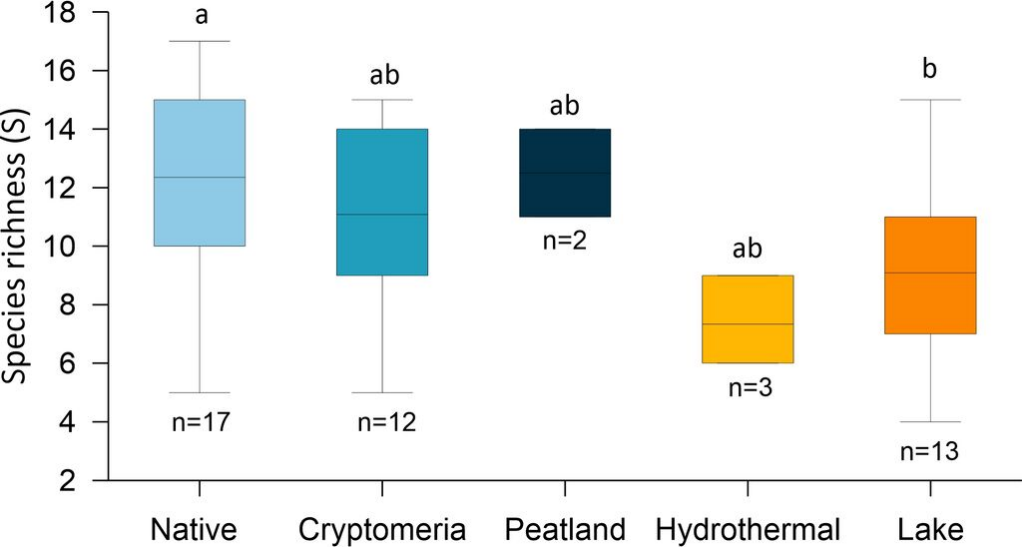
Boxplot of species richness in each sampled habitat. Different letters indicate significant differences (Tukey Test, p < 0.05).

A total of six taxa occurring at only one sampling site were considered rare (Fig. 5). These included *Cryptodifflugia oviformis*; *Euglypha filifera*; *Physochila griseola*; *Planocarina carinata*; *Pyxidicula cymbalum* and *Trachelocorythion pulchellum*. Another 14 taxa were considered occasional, occurring in two to five sampling sites. These included species, such as *Alabasta militaris*, *Argynnia dentistoma*, *Cyclopyxis kahli*, *Argynnia caudata*, *Corythion constricta*, *Difflugia bacillifera*, *Difflugia elegans*, *Euglypha acanthophora*, *Arcella catinus*, *Centropyxis constricta*, *Cyphoderia ampulla*, *Euglypha cristata*, *Padaungiella tubulata* and *Trigonopyxis arcula*. Species richness significantly differed amongst habitats (one-way ANOVA, P < 0.05). Overall species richness was in the order Natural forest > Peatland > Cryptomeria forest > Lake > Hydrothermal, but significant differences were found only between Native forest and Lake habitats (Fig. 6).

### Discussion

There are very few inventories of testate amoebae in the Azores, therefore the overall species richness of testate amoebae is unknown. Here, we present the first systematic study that explored the distribution of testate amoebae in bryophyte communities mainly in forest habitats. Forty-three species are recorded for São Miguel Island, 38 of these being new records for the Azores Archipelago. However, we must take into account the numerous cryptic taxa that testate amoebae present and some taxonomic uncertainty (Roland et al. 2017). For example, amongst Arcellinid, the *Nebela tincta*–*bohemica*–*collaris* species complex is a problematic group having very similar species (Heal 1964, Gilbert et al. 2003, Kosakyan et al. 2013). It is possible that what we identify here as *Nebela collaris* s.l. may include several taxa and, for this reason, it appears as the most abundant species. Another genus with similar difficulties is *Quadrulella* (Kosakyan et al. 2016). Considering the complexity of these groups, more detailed taxonomic work and more morphometric studies, combined with genetic approaches, such as the molecular barcoding effort, are needed to characterise this species complex.

The three most representative families in terms of species richness, Centropyxidae, Euglyphidae and Trinematidae, are, in general, the most commonly registered in other oceanic archipelagos (Smith 1986, Smith 1992, Beyens et al. 1990, Mieczan and Adamczuk 2015, Golemansky 2016) and in other parts of the world (Beyens et al. 1986, Bobrov et al. 1999, Mazei and Belyakova 2011, Acosta-Mercado et al. 2012, Šatkauskienė 2014). Considering the species richness for different testate amoebae genera in the distinct habitats studied, *Euglypha* and *Trinema* were the most frequent, followed by *Corythion* and *Centropyxis* (*Fig. 5*).

The 43 taxa recorded to the Azores is higher than what was reported to other oceanic archipelagos, such as the Canary Islands (10 species), Balearic Islands (15) and Island of Annobón (30 species). Testate amoebae assessment in the Balearic and Canary Islands was focused on mosses under *Pinus* forests of drier characteristics (Gracia 1965a, Gracia 1965b), while on the Island of Annobón (Equatorial Guinea), the work was performed on forest epiphyte mosses (Gracia 1963). However, these numbers cannot be used to draw conclusions about testate amoebae species richness in each archipelago since sampling efforts, habitats and approaches were different. In this context, it is essential to increase the sampling effort on other archipelagos, as well as to survey multiple habitats in order to find a greater diversity of testate amoebae.

The testate amoebae assemblages in São Miguel Island were composed mainly by genus with a cosmopolitan distribution which are also known from other oceanic islands. For example, in the comparable Annobón tropical rainforest, situated much further south (Gracia 1963), the diversity of testate amoebae is very similar (share 20 species). The cosmopolitan character of testate amoebae assemblages is also found in the mainland counterparts: the tropical mountain rainforest in Ecuador presented taxa geographically widespread with only nine species (6.7%) being considered tropical (Krashevska 2009). However, these biogeographic conclusions may be biased, on the one hand because of the number of cryptic species that exist and, on the other hand, because of the lack of habitat diversity surveyed. The information regarding protist communities cames from *Sphagnum* moss on peatlands (Glime 2017, Lamentowicz and Mitchell 2005). Only a few studies were made in other types of bryophytes assemblages, mainly in northern areas including Devon Island (Beyens et al. 1990), Greenland (Beyens et al. 1986), Russia (Mazei and Belyakova 2011) or in sub-Antarctic areas (Smith 1992), such as Adelaide Island (Smith 1986), South Shetland Islands (Golemansky 2016), King George Island (Mieczan and Adamczuk 2015). The biogeographical situation of the Azores Archipelago between the Nearctic and the Palearctic offers unique possibilities to study the distribution of these organisms and their ability to colonise islands. This is the case of *Argynnia caudata* present in the Azores and with a tropical/subtropical distribution.

Moss biotopes are very abundant in the Azores, where extant bryoflora comprises about 430 species of mosses and hepatics (Sjögren 2003). The subtropical forest from the Azores is more or less constantly humid and warm and supports a very rich assemblage of moss species, including a higher proportion of endemic species (Sjögren 2001). The highest species richness of testate amoebae (n = 17, 40 species) occurred in native forest habitats and corresponds to epiphytic bryophytes that grow abundantly on the bark of living trees/shrubs. This alliance Echinodion prolixi Sjn. 93 is established in part of the native forest-phytocoenoses at high altitudes (600 m), dominated in the tree-layer of *Laurus*, *Erica*, *Juniperus* and *Ilex* (Sjögren 2003). In these epiphytic bryophytes, belonging to genera *Frullania or Scapania*, the most frequent species associated with these mosses were *Corythion dubium*, *Centropyxis aerophila* and *Euglypha rotunda.* Although, native forest communities are highly degraded in São Miguel, where the best-preserved area corresponds to the high eastern part of the Island (Fig. 1, Loc.: 14, 15 and 16) and in many places have been replaced by lowland shrub and peatlands (Fig. 2, habitats 1 and 5). In fact, peatland habitats can be considered in the Azores as an extension of these wet mountain forests, where the more abundant mosses are *Polytrichum juniperinum* Hedw. and *Sphagnum* spp. Despite that, they only share 42.5% of testate amoebae species, especially from genus *Euglypha* and a lower species diversity was observed (n = 2, 17 species). However, these results must be regarded with caution, because of the low number of replicates collected and analysed from peatlands.

Most of the natural vegetation of the Island has been replaced by pastures and *Cryptomeria* forests (Fig. 2, habitat 2). These *Cryptomeria* forests are the third most sampled habitat (n = 12, 31 species). Despite being conifer monocultures, the ecotone areas maintain a similar diversity of testate amoebae, when compared to native forest and it is easy to find native bryophyte communities, such as the case of *Breutelia azorica* (Mitt.) Cardot. The most common species of bryophyte *Leucobryum juniperoideum* grows in very dense, glaucous green, swollen cushions or hummocks. Some hummocks in woodland can be massive and colonised by other bryophytes and vascular plants. This eosinophilic moss which grows mainly at the base of *Cryptomeria* trunks, due to its dense growth structure, constitutes a favourable habitat for a high diversity of testate amoebae. In fact, this moss *Breutelia azorica*, presents the most diverse assemblages of testate amoebae within the Cryptomeria forest. Species from *Nebela collaris* complex (60%), *Euglypha strigosa, Assulina muscorum* and *Trinema lineare* dominated in cushion moss *Leucobryum.* The testate amoebae communities that are richest in species are those that develop on pleurocarpic mosses and Jungermannialian hepatics in forest habitats (Figs 5, 6).

The most frequent genera shared on these terrestrial and semi-terrestrial habitats are *Nebela* and *Euglypha*, which are less represented in the aquatic systems. According to Lansac-Tôha et al. (2007), these genera possess fragile shells, which limit their occurrence in more dynamic environments, especially lakes and hydrothermal vents. It is possible that these are eurytopic species or that they are more abundant because they have greater access to their food resources in these terrestrial habitats.

Bryophytes assemblages on lake shores presented a high diversity of testate amoebae (n = 13, 33 species). Acarcarpic mosses, like *Fissidens* or *Campylopus* in more open areas near lakes, maintain less developed testate amoebae communities (Fig. 6). However, the genera *Sphagnum* and *Rhytidiadelphus squarrosus*, especially in more waterlogged areas, maintain rich testate communities (n = 13, 33 species). Several works consider aquatic environments and sediments to be the preferred habitat for these organisms, yet they end up functioning as a data collector for the surrounding ecosystems and many species are actually terrestrial (Glime 2017). In order to better understand this issue, it is important to further study species’ autoecology and habitat preferences.

On hydrothermal vents, lower diversity of testate amoebae was observed (n =3, 14 species). These vents develop an abundant bryophyte extension (Fig. 2), characterised by species that tolerate extreme conditions, such as *Sphagnum* spp, *Calliergon* sp. and *Polytrichum juniperinum*. The most frequent species are *Quadrulella symmetrica* and *Trinema lineare*. One possible explanation to this fact could be explained by their shell shape and composition, which are stiffer and more resistant, allowing their presence and permanence in this extreme habitat.

### Final remarks

Here, we presented the first study that explored the distribution of testate amoebae of different habitats from the Azores Archipelago, mainly in São Miguel Island. Moreover, this work indicates that there are typical species on the different sampled habitats. This a matter of concern on islands, where large areas of native forest have been replaced by exotic forest and changes in land uses, driven by human activities, will affect population dynamics. In order to better understand the complexity of these habitats, population dynamics and species specificity need to be carried out. Larger datasets located in different islands and habitats are required to better understand how these communities respond to environment changes. Additionally, molecular barcoding is a useful tool, not only for species identification, but also for studying evolutionary and ecological processes. The results of this study provide indications that testate amoebae assemblages are habitat specific and therefore constitute a promising group for paleoenvironmental reconstruction of Azorean ecosystems. Future studies in drier ecosystems, coastal areas and hydrothermal zones may reveal and offer us a greater diversity of these organisms.

## Figures and Tables

**Table 1.** Historical records of testate amoebae from Azores Archipelago

Taxa (Current name)	Ehrenberg 1854	Archer 1874	Gerne 1888	Barrois 1896	Gadea 1979
*Arcella vulgaris* Ehrenberg, 1830				X	
*Centropyxis aculeata* (Ehrenberg, 1830) Stein, 1857		as *Echinopyxis aculeata*	X	X	
*Centropyxis constricta* (Ehrenberg, 1841) Penard, 1890			X	X	
*Centropyxis* sp.					X
*Difflugia acuminata* Ehrenberg, 1838		X		X	
*Difflugia globulosa* Dujardin, 1837		as *D. globularis*			
*Difflugia mitriformis* Wallich, 1864		X			
*Difflugia oligodon* Ehrenberg, 1844	X				
*Difflugia pyriformis* Perty, 1849	as *D. azorica*		X	X	
*Euglypha* sp.					X
*Euglypha acanthophora* (Ehrenberg, 1841) Perty, 1849		as *E. alveolata*		as *E. alveolata*	
*Nebela collaris* (Ehrenberg, 1848) Leidy, 1879 s.l.			X	X	
*Quadrulella symmetrica* (Wallich, 1863) Kosakyan et al., 2016		as *Difflugia symmetrica*		X	
*Trinema enchelys* (Ehrenberg, 1838) Leidy, 1879	X	as *T. acinus*	X	X	

**Table 2.** Habitat characteristics and location of the sixteen studied localities in São Miguel

**Cod.**	**Locality**	**Latitude (Nº)**	**Longitude (Wº)**	**Alt. (m)**	**Date**	**Habitat**
Loc. 1	Lagoa Azul	37.97833	-25.84638	260	04- Feb.	Lake shore with degraded aquatic vegetation
Loc. 2	Lagoa Verde	37.89056	-25.80194	260	04- Feb.	Strongly altered and degraded aquatic vegetation
Loc. 3	Lagoa do Canário	37.83540	-25.75898	755	04- Feb.	Aquatic vegetation with *Sphagnum* and *Thuidium* communities
Loc. 4	Lagoa do Caldeirão Norte	37.82340	-25.75049	775	04- Feb.	Lake shore and shrub communities rich in *Sphagnum*
Loc. 5	Lagoa do Carvão	37.82360	-25.74241	700	15- May.	Lake shore and shrub communities
Loc. 6	Lagoa da Prata	37.80652	-25.73641	520	15- May.	Peatlands with *Sphagnum* and *Cryptomeria* forest
Loc. 7	Alto da Barrosa	37.76333	-25.50056	800	27- May.	Shrub communities of *Calluna* with epiphytic bryophytes
Loc. 8	Lagoa do Fogo	37.76669	-25.48024	580	22-Jun.	Lake shore and *Cryptomeria* forest
Loc. 9	Ribeira da Praia	37.72583	-25.46822	180	05- May.	*Fissidens* communities
Loc. 10	Lombadas	37.77745	-25.46167	600	11-May.	Hydrothermal, native and *Cryptomeria* forest
Loc. 11	Lagoa de São Brás	37.79306	-25.41278	600	27-Feb.	Aquatic vegetation surrounded by *Cryptomeria* forest
Loc. 12	Lagoa do Areeiro	37.76324	-25.42743	580	13-Feb.	Aquatic vegetation surrounded by *Cryptomeria* forest
Loc. 13	Lagoa do Congro	37.75522	-25.40737	700	15-May.	Lake shore and *Fissidens* communities
Loc. 14	Ribeira do Caldeirão	37.81769	-25.26294	600	27-May.	Native and *Cryptomeria* forest
Loc. 15	Planalto dos Graminhais	37.80056	-25.23944	900	10-Jun.	Native forest and peatlands
Loc. 16	Ribeira do Guilherme	37.79972	-25.20194	580	09- March.	Native forest
